# Behaviour of under sleeper pads at switches and crossings – Field measurements

**DOI:** 10.1177/0954409717707400

**Published:** 2017-06-28

**Authors:** Louis Le Pen, G Watson, A Hudson, W Powrie

**Affiliations:** Faculty of Engineering and the Environment, Infrastructure Research Group, University of Southampton, Southampton, UK

**Keywords:** Ballasted railway track, digital image correlation, geophones, high-speed filming, sleepers, switches and crossings, track stiffness, under sleeper pads

## Abstract

Major growth in rail traffic in many parts of the world in recent years has brought railway networks close to capacity and restricted the time available for track access to carry out maintenance work without costly temporary route closures. There are, therefore, significant benefits in designing or modifying ballasted track systems to reduce maintenance and associated access requirements. Under sleeper pads (USPs) offer the potential to extend ballasted track system life and to extend the intervals between routine maintenance. This paper presents and evaluates field measurements, made using geophones and high speed filming with digital image correlation (DIC), of the performance of a renewed section of track incorporating two switches and crossings (S&C) over a period of two years. One S&C was fitted with two types of USP (categorised as medium and soft), while the other had no USPs and acted as a control. Measurements demonstrate that the bearers with USPs fitted showed less variability in movement than bearers without USPs fitted. The provision of soft USPs caused large increases (>40%) in vertical bearer movements relative to bearers without USPs, although the medium USPs showed little difference. Increased movements of elongated bearers supporting both tracks fitted with soft USPs led to increased bearer rotations towards the loaded track. This effect was aided by the rigid steel collar fixing in the middle of the bearer used in this design of S&C, and raises questions concerning the desirability of this feature. DIC measurements showed that the at rest position of the elongated bearers rotated towards the track on which a train had most recently passed.

## Introduction

Many countries have seen major growth in rail traffic over the past decade or so, which has brought existing networks close to capacity. In the UK, for example, the number of passenger kilometre travelled annually is now greater than at any time in the last 60 years. Over the same period, the size of the UK rail network, currently 30,000 km of main line track, has halved. Increased network use restricts the time available for maintenance without costly temporary route closures. Night-time windows during which time rail traffic was historically absent and maintenance work carried out are becoming short and few, and on some routes non-existent. Thus, the economic case for reducing maintenance and the associated track access requirements is clearer than ever. Under sleeper pads (USPs) offer one potential approach.

Improvements in track performance from USPs can be attributed to two mechanisms that reduce maximum stresses in the trackbed. First, USPs reduce the effective rail support stiffness. This results in a spreading of the deflection bowl from an individual load along a greater length of track, and hence a lower maximum stress in the supporting trackbed. Secondly, USPs are substantially more compliant than concrete. Thus, the placement of a USP at the sleeper or bearer^a^ to ballast interface reduces grain contact stresses and increases the number of contacts between stone ballast grains and concrete bearer, reducing the potential for damage. Furthermore, the addition of a controlled compliance would be expected to result in a more homogeneous track support stiffness, reducing the dynamic loads arising from unwanted variations in support stiffness along the track.

The benefits of USPs in reducing maximum trackbed stresses can be described qualitatively and demonstrated theoretically for individual load cycles using simple closed-form equations and numerical modelling. However, real track behaviour is more complex and controlled experiments in the field are difficult to carry out on a modern, heavily trafficked railway. This paper presents and evaluates field measurements of the performance of USPs installed on a renewed section of track incorporating S&C to develop an improved understanding of how USPs change track performance and may help to reduce the required frequency of maintenance.

## Background

The Union International des Chemins de Fer (UIC) summarised USP use and trials by a number of European rail authorities.^1^ Commentary and data provided by these authorities supported the general observations that USPs can

1. compensate for localised differences in track stiffness
2. reduce on-track machine maintenance
3. reduce high-frequency vibrations and structure-borne noise

While these benefits were generally perceived, the different study sites described in the UIC report sometimes gave contradictory evidence. Hence, there remains a need to gather further data at sites where USPs are installed.

Paixão et al.^2^ carried out comprehensive field measurements to investigate the use of USPs at a transition zone onto a bridge. They showed that the introduction of USPs greatly increased the compliance of the track in the transition zone but, contrary to expectation, also increased the unevenness in the stiffness through the transition. They concluded that it is necessary to consider not only the properties of the USPs themselves but also the geometry and arrangement of the track in the transition zone.

Insa et al.^3^ developed a numerical model for a high-speed bridge transition that included USPs. They showed that USPs had little influence on the bridge loading but could influence the stresses within the ballast layer. Owing to the idealisations within the model, no issues with increased unevenness in stiffness through the transition were found. This is a limitation of numerical modelling; significant aspects of computed behaviour may depend on the assumptions made in setting up the model, which may then be unable to reproduce measured field performance.

Ali Zakeri et al.^4^ measured vibration adjacent to track where USPs had been installed. They showed that USPs were effective in reducing ground-borne vibration at frequencies in the range 40–80 Hz on plain line but could increase vibration at frequencies below 40 Hz.

**Figure 1.**
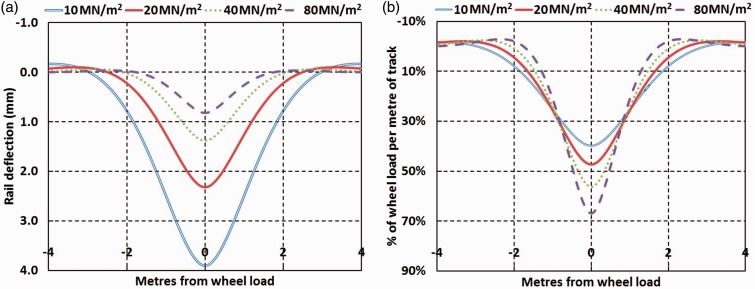
BOEF model results for (a) rail deflection for varied support moduli and a 10 t wheel load on UIC 60 Rail and (b) % of wheel load per metre transferred for different support moduli. BOEF: beam on elastic foundation; UIC: Union International des Chemins de Fer.

Assuming linear elastic behaviour, USPs can be shown to reduce the stresses transferred through the rail and bearer to the supporting trackbed, using the simple beam on elastic foundation (BOEF) model (e.g. Timoshenko^5^ and Raymond^6^). The equations can be used to determine rail deflection and the percentage of the wheel load seen by the subgrade along the rail length (per metre) for different support moduli as shown in Figure 1. As the support modulus is reduced, a greater length of the rail is mobilised in bending. This increases the maximum rail deflection but spreads the wheel load over a greater distance hence reducing its peak trackbed contact stress. This occurs because as the support modulus reduces, a greater length of bending in the rail is mobilised. Thus, a reduced support stiffness may be beneficial for the trackbed. However, the trackbed is only one part of the track system and the potential disadvantages of a softer support include increased rail bending and greater ranges of accelerations, velocities, and deflections of the rail and bearer. There is, therefore, a compromise to be made in specifying a softer USP such that it improves trackbed longevity without reducing the life expectancy of other track system components such as the bearers, rails and fasteners. These remarks are broadly in agreement with the findings of a numerical study carried out by Johansson et al.^7^

The BOEF model adopts a particular definition of track support system stiffness. It can be converted to an equivalent individual sleeper/bearer (or combined bearer and railpad) elastic spring support stiffness by multiplying by the sleeper/bearer spacing. This type of track support model is routinely employed in commercially available train/track interaction dynamic software packages (e.g. Li et al.,^8^ Msc Software,^9^ Deltarail^10^).

While a homogeneous support is convenient for modelling, field measurements have demonstrated that the bearer support stiffness can vary significantly, even over short lengths of track (e.g. Oscarsson,^11^ Bowness et al.,^12^ Le Pen et al.^13^ and Murray et al.^14^). Numerical simulations have demonstrated that significant additional dynamic loads occur as a result of varying support stiffness (e.g. Bezin et al.^15^). A more variable support stiffness is generally considered to cause accelerated track degradation (e.g. Hunt^16^ and Sussman et al.^17^). Variations in support stiffness may occur as a result of hard substructures such as bridges and culverts. Transitions onto and off these locations are particularly difficult to maintain (e.g. Coelho et al.,^18^ Paixao et al.,^19^ Varandas et al.^20^ and Milne et al.^21^).

**Figure 2.**
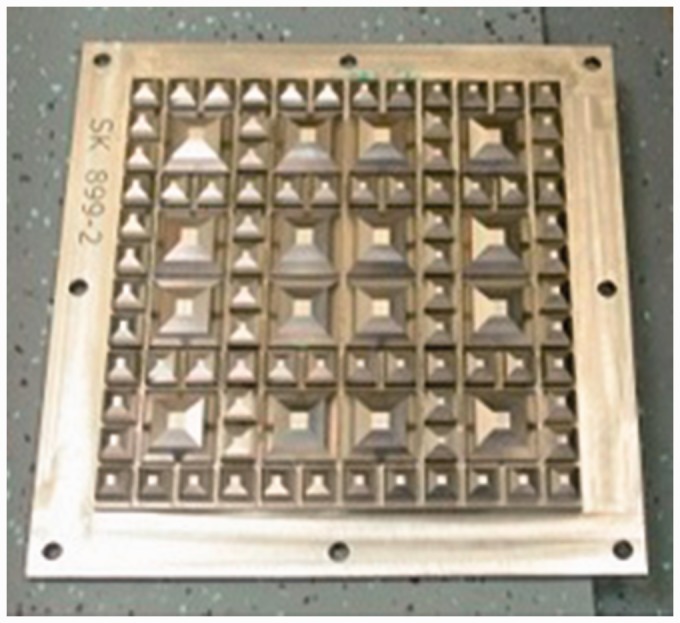
Geometric plate used to test under sleeper pad.

USPs are available from a variety of manufacturers, fabricated from a range of materials (e.g. polyurethane, rubber, Ethylene-vinyl acetate [EVA]). They may be fitted to bearers at manufacture by casting a modified roughened facing on one side of the USP into the concrete or by gluing. Standards have evolved that enable USPs to be classified and specified using a defined stiffness determined by a prescribed method (DIN 45673-6 ^22^ and BS EN 16730:2016^23^). Current procedures include load/unload tests using a geometrically patterned loading plate (Figure 2) to represent, in a repeatable way, a levelled, compacted ballast surface. For a specified stage of the load/unload sequence and a given increment of stress at a specific temperature, a static stiffness (***C_stat_***) may be determined and used to categorise USPs as shown in Table 1. Other measures of stiffness for a given loading frequency and/or different stress increments and temperatures are also used, along with a variety of other strength and fatigue tests to verify the suitability of a USP for long-term deployment.

**Table 1.** Stiffness categorisation of USPs.^24^

Category	***C_Stat_*** (N/mm^2^)
Stiff	0.25 ≤ ***C_Stat_*** ≤ 0.35
Medium	0.15 ≤ ***C_Stat_*** < 0.25
Soft	0.10 ≤ ***C_Stat_*** < 0.15
Very soft	***C_Stat_*** < 0.10

USP: under sleeper pads.

***C_stat_*** could be used to estimate the increment of deflection for a known wheel load, using the footprint area of the bearer. However, non-linearity and the uneven stress distributions beneath the bearer^25^ mean that it may be more appropriate to measure behaviour under more realistic laboratory or field conditions, accepting that there will be some variation in the data obtained.

To overcome the difficulties in relating theory and laboratory tests on isolated pads to field performance, Abadi et al.^26^ carried out tests on stiff and soft USPs having *C_stat_* values of 0.31 and 0.11 N/mm^2^, respectively, in a laboratory apparatus representing a single sleeper bay under plain strain conditions. A monoblock concrete sleeper was placed on a ballast bed 300 mm deep. Further details of the apparatus and the methods used are available in Le Pen and Powrie^27^ and Abadi et al.^28^ Tests over three million cycles of a 20 t equivalent axle load at 3 Hz showed that the USPs increased the resilient range of movement and reduced the rate of permanent settlement. After one million cycles, the resilient deflections were 0.08 mm greater with the stiff USP and 0.59 mm greater with the soft USP than for the sleeper without USP.

However, laboratory tests cannot reproduce all aspects of field behaviour. For example, in the field, individual bearers may be voided^b^ owing to the quality of construction and the evolution of differential track settlement. Variations in the support stiffness along the track disrupt the pattern of load distribution from the ideal uniform case and may result in increased stresses on and within the trackbed. Trains with different dynamic characteristics travelling at different speeds will cause different dynamic components of load. Thus, behaviour observed in the laboratory cannot be a direct predictor of field performance. Nevertheless, the laboratory data may be used to relate the additional movement of a USP-supported sleeper or bearer (***δ_USP_***) to the range of ***C_stat_*** tested (0.11–0.31 N/mm^2^) as: ***δ_USP_*** = 0.87−2.55.***C_stat_*** for a 20 t axle load.

Measurements in the same tests using pressure sensitive paper^26^ showed that the introduction of USPs increased the number of ballast to sleeper contacts from 147 for a non-USP test to 314 and 447 for the stiff and soft pads tested, respectively. For a logarithmic particle size distribution and an idealised cubic packing, it was estimated that the use of the USPs had increased the proportion of potential contacts actually realised from 20% in the non-USP case to 61% and 87% for the stiff and soft USPs, respectively,^26^ thus quantifying aspects of the previously qualitatively described expected beneficial behaviour.

## Field measurement methods

Various methods are available to measure track movements. These include lasers,^2^ multidepth deflectometers,^29^ accelerometers,^30^ high-speed filming with digital image correlation (DIC)^12,14^ and geophones.^18,31^ For this research, geophones and high-speed filming with DIC were selected owing to their versatility and complementary capabilities. A comparison of the different methods available and their relative advantages and disadvantages is given in TSWG.^32^

**Figure 3.**
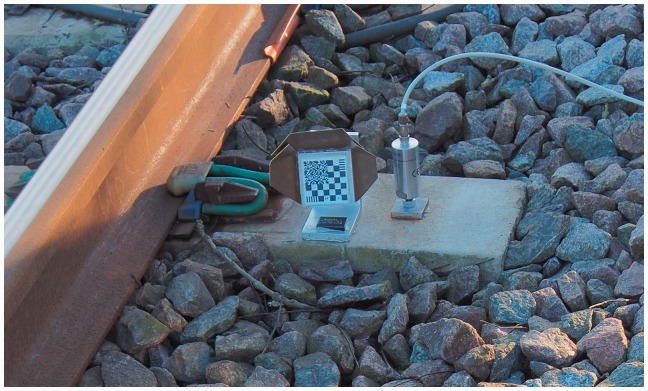
Geophones and digital image correlation target.

The use of geophones to measure bearer movements as trains pass has become established practice. Geophones are small velocity transducers. They can be fixed to a bearer and connected by cable to a data logger to record bearer movement velocities as trains pass (Figure 3). The raw measured voltages are converted to deflections by applying an appropriate calibration, filtering and integrating. The displacement range for individual axle passes can be obtained from the time/displacement trace. A summary of geophone-based measurement and interpretation is given in Le Pen et al.13,^33^ Le Pen et al.^13^ provide detailed information of the same data acquisition system (acquisition rate 500 Hz) and geophones used for this study.

A complementary method of obtaining track movements is the use of high-speed video and DIC (e.g. Bowness et al.^12^ and Murray et al.^14^). A target may be attached to a bearer (Figure 3) and videoed; sometimes the target can be omitted and the texture of objects, such as the rail web, tracked. Analysis is carried out using a variant of the DIC technique described by White et al.^34^ and Bhandari et al.^35^ The technique involves identifying corresponding patterns in successive images using a normalised cross-correlation algorithm. There are two major sources of noise in DIC resulting from camera movement: (1) ground-borne and (2) air-borne (train slip-streams and wind) vibration. Both tend to increase with train speed. Noise mitigation techniques are described by Le Pen et al.^13^ and Wheeler et al.^36^

## The field study site

### Layout

**Figure 4.**
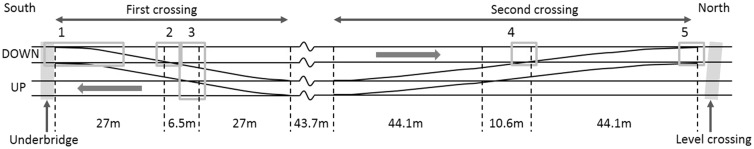
Schematic plan of the study site indicating instrumented zones labelled 1–5.

The study site is approximately 250 m in length and comprises two tracks, labelled the Up and Down^c^ main lines (Figure 4). Two track crossings are present, but for trains not utilising the crossings the maximum operating speed is 177 km/h (110 mile/h). Instrumentation was located mainly on the Down line, with some on the Up. Instruments were located within the numbered rectangles (1–5) in Figure 4, centred on switchblade tips and crossing noses and are described in more detail later. On the Down line, trains approach the study site over an underbridge; immediately after the underbridge, facing switchblade tips enable trains to cross over to the Up line. Further along, trailing switchblade tips enable trains to rejoin the Down line. Just beyond the end of the site is a level crossing. Data reported are from trains travelling at or near the line speed.

The angle of the first crossing nose is 1:13 and of the second is 1:21. The distances between switchblade tips and crossing noses are shown in Figure 4. Twist rails transition the rail from leaning inward to vertical over each crossing. Bearers are concrete type G44 spaced at 650 mm centres on the plain line and type 001 for the length of the crossover, with six type 001E leading onto the crossing and three type 001E exiting the crossing. The type 001 E bearer enables the transition from the G44 to the 001 bearer. The gauge of the track is 1435 mm except through the crossing where it is 1432 mm. The rails are type CEN56E1 and CEN56E1A1 (vertical).

The crossing construction includes long concrete (type 001) bearers made up of two parts, joined together using steel collars and bolts (see Figure 9(b)). These elongated bearers tie the two main lines together, with the aim of preventing differential lateral movement between them through the crossings. The use of steel bolted collars enables the long bearer to be transported on conventional road and rail vehicles in two standard size sections, which are then fixed together on site. The design is approved for use on the UK network and is intended to provide six degrees of restraint (moment and force in three orthogonal directions) at the joint. However, other designs are used in other rail authority regions; in some cases restraint is provided by pinned connections that permit rotational movements between adjacent tracks. The railway at this location is on an embankment, whose height above the local topography varies from about 4.0–4.5 m at the south (underbridge) end to 2.0–2.5 m at the north end of the site.

**Figure 5.**
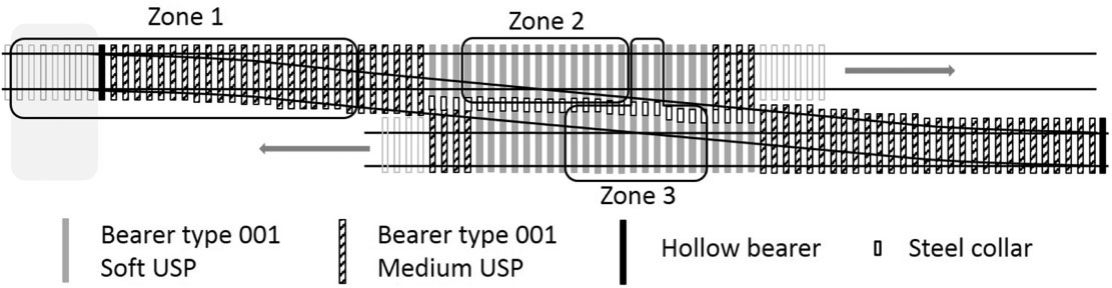
Plan of individual bearers fitted with under sleeper pads at the first switch and crossing and the locations of zones 1, 2 and 3.

**Figure 6.**
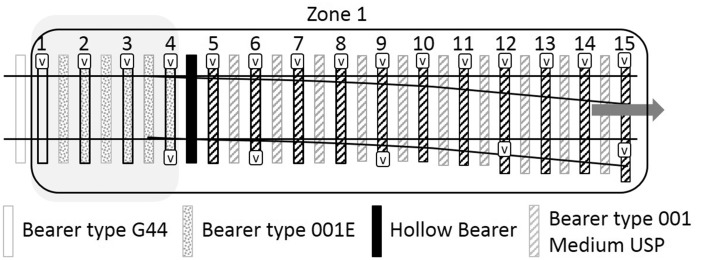
Zone 1 instrumented bearers numbered in travel direction (V = vertical geophone).

**Figure 7.**
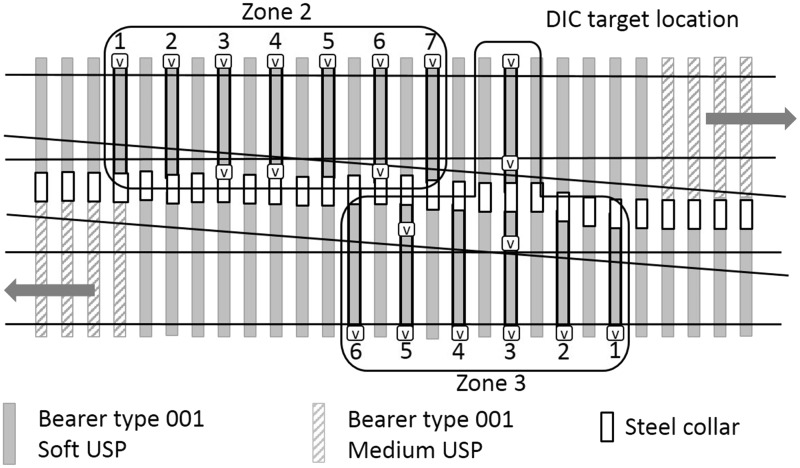
Zones 2 and 3 instrumented bearers numbered in travel direction (V = vertical geophone).

**Figure 8.**
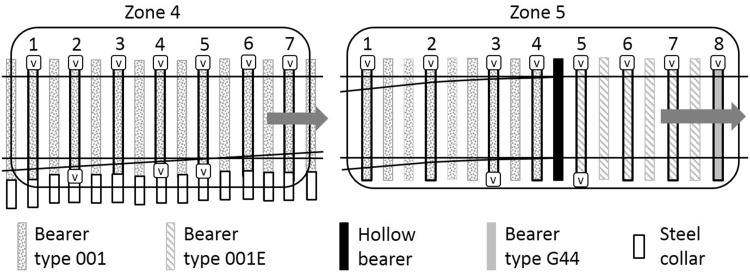
Zones 4 and 5 instrumented bearers numbered in travel direction (V = vertical geophone).

A renewal of the first crossing was carried out in December 2012. This comprised replacement of the ballast to a depth of 300 mm below the bearer soffit and replacement of the rails, bearers and other track components. During this work, USPs associated with the first crossing were fitted. Two types of USPs were specified (Table 2), with the intention of giving a controlled change in support stiffness through two steps into the S&C and then back again. Figure 5 illustrates the arrangement of USPs over the first crossing with shading to indicate the bearer and USP type in zones 1, 2 and 3. Close-up diagrams of all five zones are presented in Figures 6 to 8 where instrumented bearers are also indicated. USPs were placed along the full length of the joined concrete bearers; four of these bearers on each side of the crossing nose had one type of USP on one side and a different type of USP on the other.

**Table 2.** Types of under sleeper pads installed at the trial site.

	Types of USP used in site trial	
	Medium USP	Soft USP
Thickness	10 mm	10 mm
Weight	4.2 kg/m^2^	4.2 kg/m^2^
Stiffness ***(C_Stat_)*** 23℃ 0.02–0.16 N/mm^2^	0.22 N/mm^2^	0.15 N/mm^2^
Core material	Polyurethane	Polyurethane

The second part of the study site, including the second crossing, was renewed without USPs around December 2013. This latter renewal was only a skim dig, involving the replacement of just the track components and the ballast above the bearer soffit (i.e. the older existing ballast below the bearer base was left in place).

The performance of the two renewed sections may be compared to evaluate the effect of the USPs. However, in addition to the different depths of ballast replacement there are some further complicating features of the study site. The crossings have slightly different lengths and crossing angles. The first crossing is preceded by an underbridge, while a level crossing is located beyond the second. Ground investigations carried out in 2011 indicated that prior to renewal the top 0.3 m of ballast below bearer soffit was intermixed with ash and degraded; this was underlain by a layer of coarse ash intermixed with ballast from approximately 0.3–0.7 m below bearer soffit giving way to coarse ash and then sand at depths greater than 1.2 m to at least 1.6 m. Below this, the core of the embankment material was not investigated.

Five zones of track (Figure 4) were instrumented by moving a set of 20 vertical geophones and two data loggers between zones, during each of a total of four, two-day long site visits in February 2014, December 2014, April 2015 and March 2016. During monitoring, all trains remained on their respective lines with neither crossing being activated. At each zone, geophones were placed for periods of up to 18 h on each visit (sometimes overnight). Zone 1 includes the medium USPs. Zones 2 and 3 include the soft USPs. Zone 4 has no USPs, is further along the Down line and is approximately comparable with zone 2 but with the crossing oriented in the reverse direction. Zone 5 (with no USPs) is the comparator for zone 1, but with the switch blade tips trailing oncoming trains. The instrumentation locations are detailed in Figures 6 to 8.

**Figure 9.**
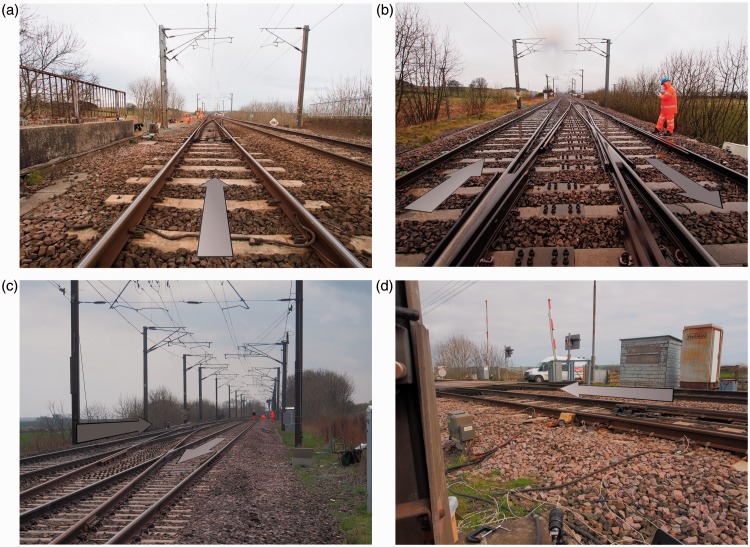
Arrows indicating train direction in (a) zone 1, (b) zones 2 and 3, (c) zone 4 and (d) zone 5.

Figure 9 shows the photographs of the study zones.

### Track recording vehicle data

**Figure 10.**
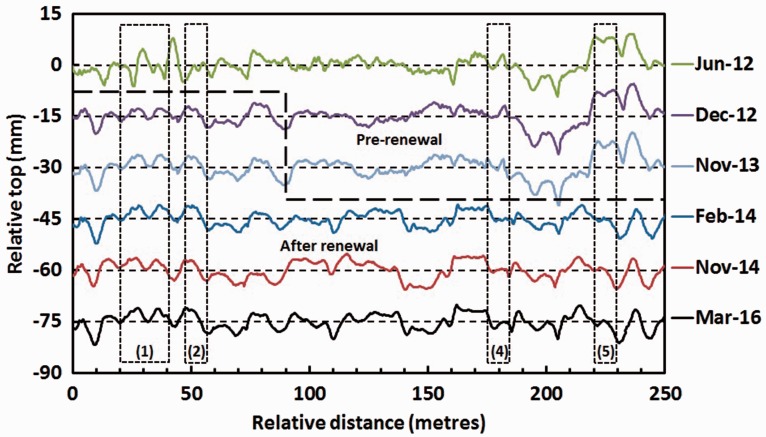
NMT data showing the vertical deviation filtered for a 70 m wavelength on the Down line.

Selected traces at key dates for the average vertical top from both rails filtered at 70 m wavelengths, obtained from the Network Rail New Measurement Train (NMT), are shown in Figure 10. For ease of comparison these are offset vertically by increments of −15 mm as dates become more recent. The estimated positions of zones 1, 2, 4 and 5 are also indicated in Figure 10.

The NMT traces in Figure 10 indicate that major changes in track performance through the crossings were associated with the renewals of the two portions of track. The June 2012 data were recorded prior to both renewals and generally show more extreme variations in top level. The first portion of track, from 0 m to approximately 100 m, was renewed and USPs installed between the June and December 2012 traces; the December 2012 trace indicates the improvement in performance after this renewal. The second portion of track, from approximately 100 to 250 m, was renewed between the November 2013 and February 2014 traces; the February 2014 trace suggests that this section was less obviously improved and in places appears more variable, perhaps because the renewal was less comprehensive. The traces after each renewal indicate that generally relatively minor changes in support conditions are continuing.

**Figure 11.**
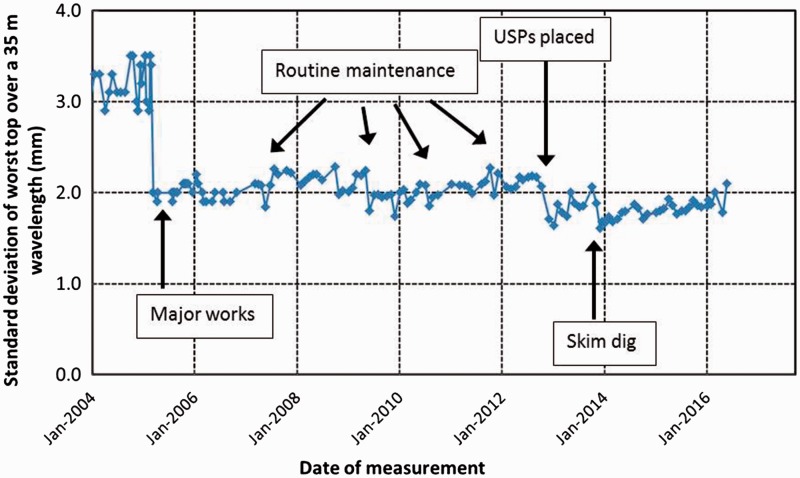
Standard deviation for the worst 35 m wavelength vertical top between 20 and 220 m, Down line.

Further insight into the track quality can be obtained from the graph of standard deviation (SD) of the vertical level of the track against time, shown in Figure 11 for the worst vertical top from both rails filtered at a 35 m wavelength on the Down line. Following UK convention, the SD is calculated per 1/8 of a mile (approximately 200 m); the nearest 1/8 of a mile for which data are available corresponds to the length 20–220 m shown on the x-axis in Figure 10.

In Figure 11, abrupt changes in SD coincide with track interventions such as renewal and tamping (e.g. the major reduction in SD in 2005). The effects of the ballast renewal with USPs (December 2012) and the skim dig of the second crossing (December 2013) are apparent. The current track quality (SD value) appears to have been improved by these interventions, although some variation in the SD data may be attributable to a lack of exact alignment of successive measurement train runs and to seasonal effects.

## Field monitoring results

A number of different vehicle types and train configurations pass the study site. For clarity, data are presented for only one vehicle type – a Class 91 locomotive – and for the movements measured during the passage of the third axle only of this four-axle locomotive. This vehicle was selected because high-quality records were obtained for a number of passes at each zone. The axle load is also more consistent than freight and passenger carrying vehicles, for which the live load is more variable. The static axle weight of the Class 91 is 20.4 t, which is amongst the heaviest regularly traversing the track. In 2014, the Down line carried the equivalent of 15 million tonnes (15 equivalent million gross tonnes per annum), roughly 20% of which was freight and 80% passenger traffic.

The measurements were taken over the four visits encompassing 25 months of track use, corresponding to approximately 31 EMGT of traffic.

**Figure 12.**
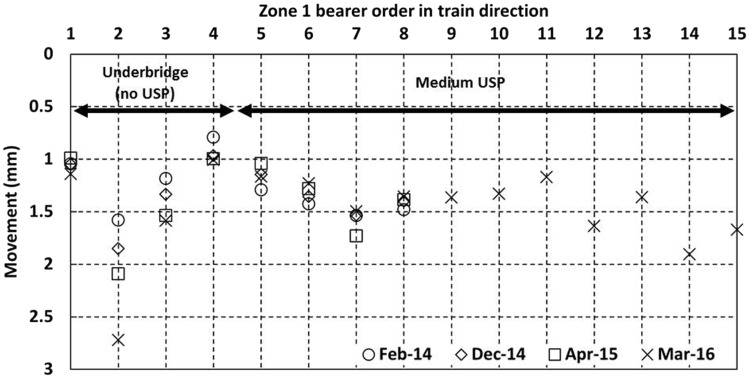
Vertical movements for cess end of bearers in zone 1 as trains pass on the Down line.

**Figure 13.**
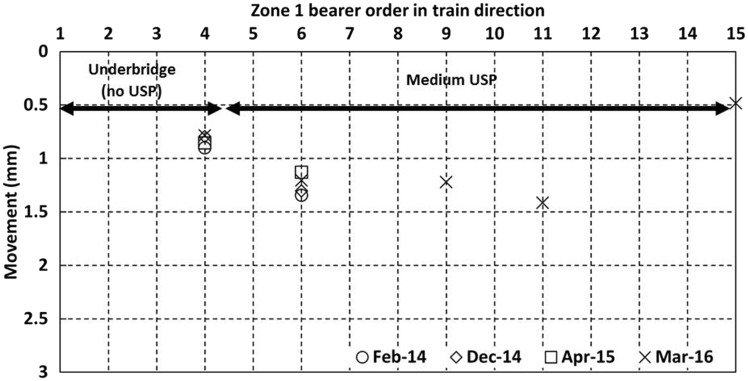
Vertical movements for 6 ft ends of bearers in zone 1 as trains pass on the Down line.

Figures 12 and 13 show data for the cess^d^ and 6 ft bearer vertical movements, respectively, as trains passed on the Down line in zone 1 from each of the four visits. Bearers 9–15 were only instrumented on the final visit but bearers 1–8 were instrumented on each visit. The movements at each visit were similar, with each measured bearer location moving between 1 mm and 2 mm. However, there are exceptions. The movement of the cess end of bearer 2 on the bridge increased at each visit to 2.7 mm, consistent with the development of voiding. Bearer 4 showed lower movement than most bearers and this was reproduced on both sides of the track. The 6 ft end of bearer 15 showed particularly low movement of only ∼0.5 mm, perhaps in part due to its extended length at the 6 ft end as it supports the switch blade (Figure 6). There is little to distinguish the average magnitude of vertical movements of the on-bridge bearers (1–4, without USPs) from those of the off-bridge bearers with the medium USP support (5–15). The greater and increasing movement at bearer 2 may be indicative of dynamic train/track interaction at the bridge. Five bearers (4, 6, 9, 11, 15) were instrumented at the 6 ft end; these measurements show that on average both ends moved by similar amounts although there could be localised variation, e.g. the large difference between the movements at either end of bearer 15 (1.6 and 0.5 mm).

**Figure 14.**
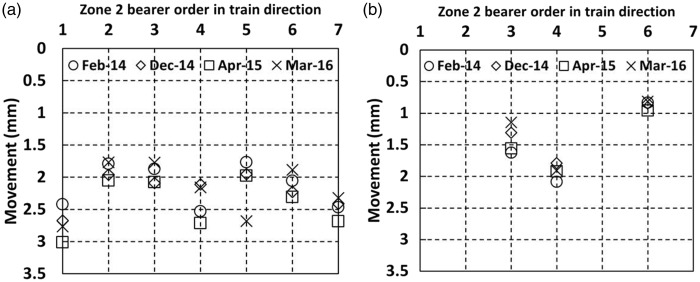
Movements for bearers at zone 2 with soft under sleeper pads as trains pass on the Down line (a) cess end and (b) 6 ft end.

Figure 14 shows movements in zone 2 as trains pass on the Down line, for (a) the cess end, and (b) the 6 ft end of the portions of the elongated bearers supporting the Down line.

In zone 2, the soft USPs are fitted to elongated bearers that stretch across both main lines on either side of the crossing nose located on bearer 4. Figure 14(a) shows cess side vertical movements between 1.7 mm and 3 mm; this is generally substantially greater than the movements in zone 1, where no USPs or the medium USPs were fitted. There is some variation between visits, with movements at an individual bearer location either increasing or reducing with no consistent trend. The bearer at the crossing nose (bearer 4) seems to behave similarly to other bearers. The three bearers on which measurements were taken at the inner (6 ft) ends moved significantly less than outer (cess) ends, suggesting that as trains pass on either side, the long bearers experience a rotational movement towards the loaded track. The ratios of cess to 6 ft end movements were 1.5, 1.1 and 2.3 for bearers 3, 4 and 6, respectively, during the April 2015 visit. The higher ratio at bearer 6 is perhaps a consequence of particularly uneven support conditions along this bearer.

**Figure 15.**
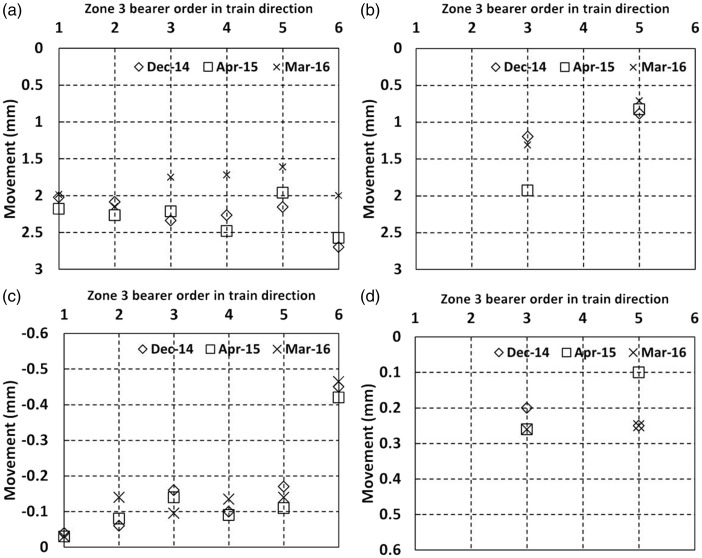
Movements of bearers in zone 3 (a) Cess end, trains on the Up line (b) 6 ft end, trains on the Up line (c) Cess end, trains on the adjacent Down line (d) 6 ft end, trains on the adjacent Down line.

Figure 15 shows the vertical movements in zone 3, where instrumentation was largely located on the Up line as trains passed on both the Up (a and b) and Down lines (c and d). In Figure 15(a) cess end movements were between 1.5 mm and 2.7 mm downward as trains passed immediately above. In Figure 15(b) movements of between 0.6 mm and 2 mm occurred on the 6 ft ends as trains passed on the Up line immediately above. In Figure 15(c) the cess ends of the Up line ***lifted*** by between 0.02 mm and 0.45 mm as trains passed on the adjacent Down line and in Figure 15(d), 6 ft ends of the Up line moved a more modest 0.1 mm to 0.3 mm as trains passed on the adjacent Down line. These measurements as trains passed on both tracks indicate that the rotational motions of bearers are severe enough to cause uplift on adjacent track at the unloaded bearer ends.

**Figure 16.**
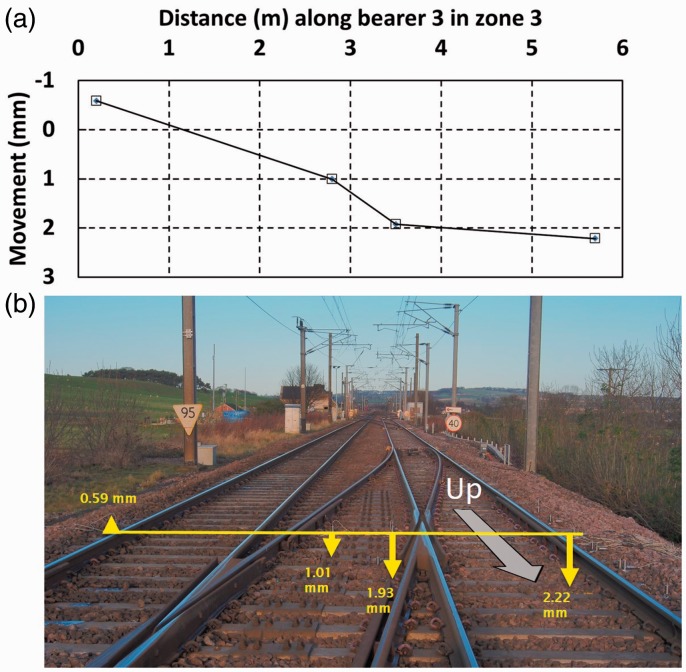
Long bearer behaviour at zone 3 from April 2015 visit as a train passes on the Up line (a) graph and (b) pictorial representation.

Bearer 3 was instrumented at four locations over its length across both lines. Figure 16 illustrates the behaviour of bearer 3 in zone 3 as trains passed on the Up main line for the April 2015 visit. Other visit data were similar. The measured data points have been joined by straight lines, although in practice the bearer movement will have been less linear, probably with slight rotations at the fixings for the steel collar joining the bearer together in the middle. The measurements show that the whole track is rocking as trains pass. Such behaviour is likely to contribute to increased rates of track geometry deterioration.

**Figure 17.**
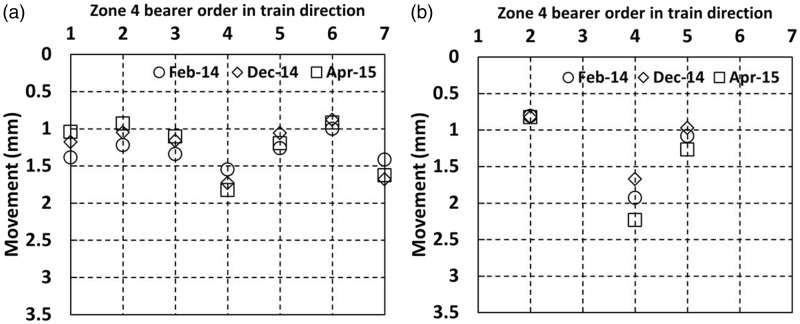
Movements for bearers without under sleeper pads in zone 4 as trains passed on the Down line (a) cess end and (b) 6 ft end.

**Figure 18.**
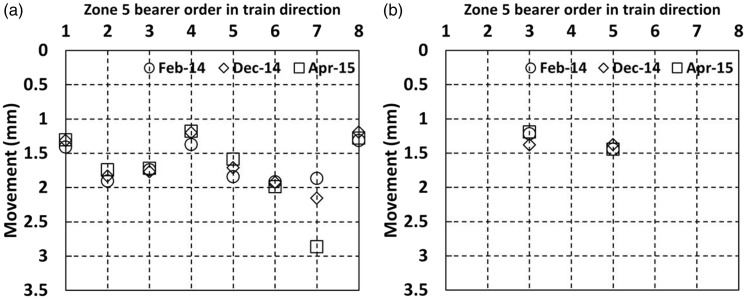
Movements for bearers without under sleeper pads in zone 5 as trains passed on the Down line (a) cess end and (b) 6 ft end.

Figures 17 and 18 show (a) the cess end and (b) the 6 ft end movements of the instrumented bearers in zones 4 and 5 as trains passed on the instrumented Down line.

Bearer movements at all locations within zone 4 (Figure 17) were between 0.8 mm and 2.3 mm. This is considerably less than in zone 2 (Figure 14), where soft USPs were installed. Both zones 2 and 4 include the position of a crossing nose, located at the bearer labelled 4 in each case. The ratio of the cess to 6 ft movements of the bearers in zone 4 is close to unity: 1.1, 0.8 and 0.9 for bearers 2, 4 and 5, respectively, during the April 2015 visit. This may suggest that lower overall magnitudes of movement are less likely to be associated with rotation of the bearers.

Figure 18 shows the movements in zone 5, with the trailing switchblades intended to be a comparable location to the facing switchblades in zone 1. The movements in zone 5 (no USPs installed) were between 1.2 mm and 2.8 mm. There is no obvious difference between the movements at the cess and 6 ft positions. Overall, the movements in zone 5 were similar to those of zone 1 (Figures 12 and 13), with no clear additional contribution from the medium USPs to the movement of the bearers in zone 1.

**Figure 19.**
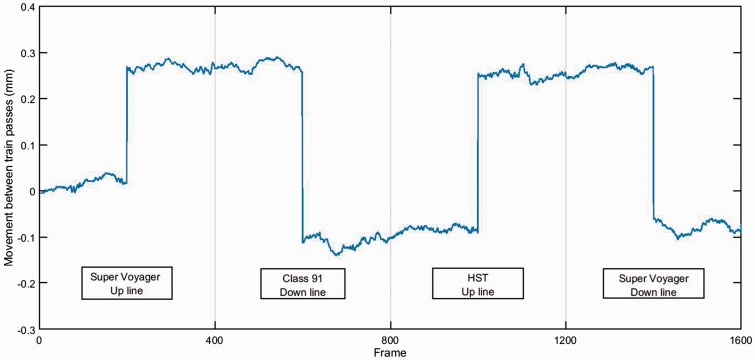
Movements for cess end of bearer 3 in zone 3 between trains passing on each line.

High-speed filming using targets (Figure 3) was carried out for a number of bearer ends where geophones were also present, and the images evaluated using DIC to confirm the reliability of the geophone measurements. Because the high-speed filming and DIC results largely duplicate the geophone data, most are not reported. However, an advantage of high-speed filming with DIC is that the absolute movement of the track relative to its initial level is recorded. This can be used to illustrate a further mechanism of behaviour for this design of S&C. Figure 19 shows the at-rest position of the Down cess end of bearer 3 in zone 3 before and after trains have passed relative to its initial level. Bearer 3 is a long bearer passing beneath both the Up and Down lines and joined in the middle with a steel collar and carries the Up line crossing nose (Figure 7). High-speed video was recorded at 250 frames per second (fps) for some 20 s capturing video prior to, during and after each train pass. In Figure 19, the first and last 200 frames from each of four consecutive train passes are plotted omitting the frames during which the train passed. The vertical line joining the first and last 200 frames within each of the four labelled sections of Figure 19 represents the net movement of the bearer end caused by the train pass. The level at the end of each train pass is approximately the same as at the start of the next train pass. Figure 19 shows that the at-rest level of the Down cess end of bearer 3 rises by 0.3 mm to 0.4 mm after a train passes on the Up line and falls by the same amount after a train passes on the Down line. The time interval between train passes was typically 15 to 20 minutes. The overall time from the first train pass to the last shown in Figure 19 was 55 minutes. There is some fluctuation in the at-rest levels between each train pass. This was caused by ground-borne vibration and/or wind from passing trains influencing the camera position. These fluctuations are at least an order of magnitude smaller (∼0.02 mm) than the more substantial movement between trains passing (>0.3 mm).

## Discussion

The measurements of bearer vertical movement can be categorised with reference to the support conditions. There are measurements from up to 24 bearers without USPs, of which four are above the underbridge, up to 12 bearers supported by the medium USPs and up to 18 bearers supported by the soft USPs. Some bearers had two or more geophones located on them at positions along their length. Not all bearers were monitored at every visit; zone 3 was omitted from the first visit and zones 4 and 5 omitted from the final visit although zone 1 was extended. These changes were made due to shortfalls in time available or because instrumentation was focused on improving the understanding of particular aspects of behaviour. Table 3 shows the average vertical movements for each visit, categorised by support condition. Table 4 shows, for the USP supported bearers, the increase in deflection in millimetre and as a percentage compared with bearers without USPs. Where present, measurements from multiple locations on individual bearers are included in the averaged data but only for trains that pass directly above. The averaged data show no discernible increase in movement that can be attributed to the provision of medium USPs compared with non-USP bearers. However, this may be because of complex transitional behaviour related to the underbridge and/or because many of the non-USP bearers are from the section of track (zones 4 and 5) for which the renewal of the ballast did not extend below the bearer soffit.

**Table 3.** Average movements (mm) categorised by bearer support condition, number of measurements averaged in brackets.

Visit	1	2	3	4
Non USP	1.38 (24)	1.37 (24)	1.45 (24)	1.61 (4)
Medium USP	1.42 (5)	1.35 (5)	1.31 (5)	1.41 (12)
Soft USP	1.94 (10)	1.99 (18)	2.05 (18)	1.80 (18)

USP: under sleeper pad.

**Table 4.** Comparison of the movements of bearers with and without under sleeper pads (USPs).

	mm increase for USPs				% increase for USPs			
Visit	1	2	3	4	1	2	3	4
Medium USP	0.04	−0.02	−0.13	N/A	3%	−2%	−9%	N/A
Soft USP	0.56	0.62	0.60	N/A	41%	45%	42%	N/A

For the bearers supported by the soft USPs the data provide clearer results. Table 4 indicates a greater than 40% increase in deflection compared with the non-USP bearers during the first three visits (visit 4 may be discounted owing to the absence of any non-USP bearer measurements other than on the bridge). The relative behaviour of non-USP bearers on and off the underbridge is similar, with the on-bridge, non-USP bearers deflecting slightly less than their off-bridge counterparts (Table 5). The simple linear correlation ***δ_USP_*** = 0.87−2.55.***C_stat_*** based on the laboratory tests reported by Abadi et al.^26^ may be applied to the two USP types used in the field trial. The field trial USPs have ***C_stat_*** values of 0.22 N/mm^2^ (medium) and 0.15 (soft); the laboratory-derived relation therefore gives increases in deflection of 0.31 and 0.49 mm for bearers with the medium and soft USPs, respectively. The medium USPs gave no clearly measurable increase in movement in the field. For the soft USPs, the additional deflection of 0.49 mm estimated on the basis of the laboratory sleeper rig tests is comparable with the 0.56 to 0.62 mm increase in movement measured in the field bearing in mind the uncertainty in the field data associated with the rotational response of the long bearers and other features of the crossings and renewals that vary along the track.

**Table 5.** Average movements (mm) for bridge and non-bridge bearers without under sleeper pads.

Visit	1	2	3	4
Bridge	1.15	1.30	1.40	1.61
Non-bridge	1.43	1.40	1.45	N/A

**Figure 20.**
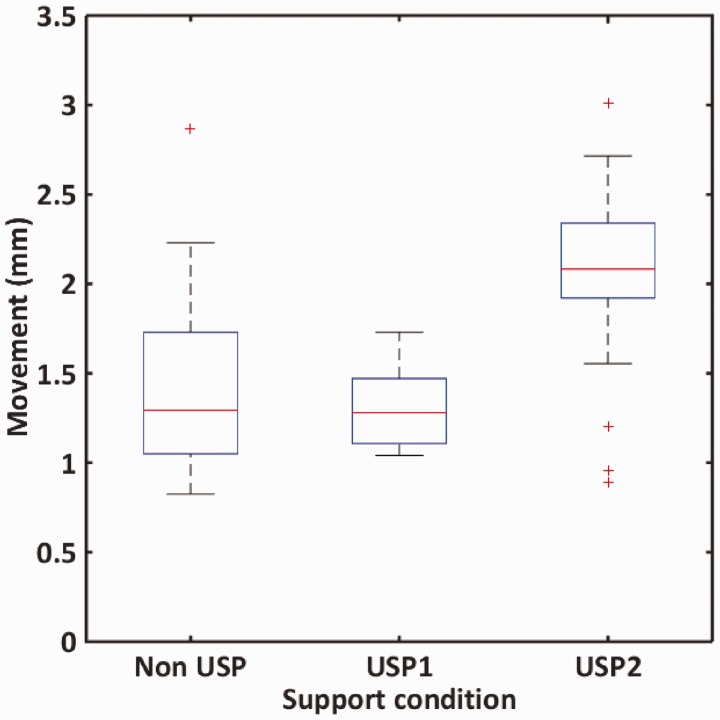
Boxplot to summarise bearer movement data for Class 91 loco.

Figure 20 shows a boxplot summary of the third visit (April 2015) data. Outliers more than the interquartile range from the upper or lower quartile are plotted beyond the whiskers of the boxplot. The interquartile range is slightly less for the soft USP than for the non-USP bearers, but the mean movement is significantly larger with an increased number of outliers. The large outlier for the non-USP bearers (with a movement of nearly 3 mm) can be attributed to under-bearer voiding. The smaller outliers for the soft USP are associated with the inner (6 ft) side of the track measurements and are a result of the rotational behaviour of the longer joined bearers as illustrated in Figure 16. The single larger outlier for the soft USP is at the first bearer in zone 2 where, in the absence of any clear on-track trigger, under bearer voiding is again the most likely explanation.

## Conclusions

This study has followed the development of track performance at a site fitted with USPs for approximately two years. Over this time, measurements taken to characterise mechanisms of behaviour enable the following observations to be made.

1. The provision of the soft USPs has caused large increases (>40%) in bearer vertical movements compared with non-USP bearers.
2. There was little difference in movement magnitudes between the non-USP and medium USP supported bearers. However, complicating features at the site (i.e. the underbridge, level crossing, changing embankment height, different crossing angles and differing renewal specifications) make direct comparison problematic.
3. For USP-fitted bearers, there was less variation in the range of movement than for bearers without USPs. Reducing the range of movements between nearby bearers should reduce variations in dynamic load.
4. Installing soft USPs on elongated bearers supporting both tracks exacerbated the tendency for these long bearers to rotate towards the loaded track as a train passes. This behaviour is also a consequence of the rigid steel collar fixing in the middle of the bearer used in this design of crossing, which raises a concern about the use of this feature. The at-rest position of the track varied remaining rotated towards the track on which a train had most recently passed.

The current general quality of the section of track as characterised by the SD for the worst top for the 35 m filter over the relevant 1/8 of a mile of track or approximately 200 m (Figure 11) is consistent with good to satisfactory track that is unlikely to require maintenance in the near future.

## Notes

a The term *sleeper* refers to a standard sized bearer supporting a single railway track. The term *bearer* applies more generally and includes concrete beams used at switches and crossings (S&C) to support switch rails and tie two tracks together.

b *Voided* bearers are also commonly known as *hanging* bearers, both terms refer to the development of a gap between sleeper/bearer and ballast.

c In the UK, the terms Up and Down usually refer to the direction in relation to London (Up to London).

d When there are two tracks the term *cess* refers to a sleeper end on the outside of either line and the term 6 ft (6 foot) refers to the space between the two lines, 6 ft is not an actual measurement although 6 ft or approximately 1.8 m is the approximate distance between the two adjacent inner rails of the two lines.
